# Single‐Cell RNA and Transcriptome Sequencing to Analyze the Role of Lactate Metabolism in Traumatic Brain Injury Astrocytes

**DOI:** 10.1002/brb3.70428

**Published:** 2025-05-21

**Authors:** Zhang Bu, Yuqian Zhou, Feng Xu, Shan Xu

**Affiliations:** ^1^ Department of Emergency Medicine The First Affiliated Hospital of Soochow University Suzhou Jiangsu China; ^2^ Soochow University Campus Hospital Soochow University Suzhou Jiangsu China

**Keywords:** astrocytes, lactate metabolism, traumatic brain injury

## Abstract

**Purpose:**

After traumatic brain injury (TBI), ischemia and hypoxia of brain tissue, glucose undergoes anaerobic fermentation, leading to a large accumulation of lactic acid. Our aim was to explore the role of lactate metabolism in brain cells after TBI.

**Method:**

In scRNA‐seq dataset, 10‐week‐old male C57BL/6 J mice were randomized to undergo mild fluid percussion injury or sham surgery, and we analyzed frontal cortex tissue during the acute (24 h) and subacute (7 days) phases of TBI at single‐cell resolution. Cell cycle phases were evaluated, and principal component analysis was performed. Cell populations were identified and visualized using the UMAP downscaling technique. Differentially expressed genes (DEGs) were analyzed using the “FindAllMarkers” algorithm. In addition, the set of genes related to lactate metabolism was evaluated using the AUCell score. GO and KEGG enrichment analyses were performed to investigate the functional pathways of DEGs in astrocytes in the acute and subacute phases of TBI.

**Results:**

A total of 13 cell populations were distinguished, including neurons, astrocytes, and oligodendrocyte progenitors. The number of neurons, astrocytes, and endothelial cells was reduced in the TBI group compared with the sham group. During the acute phase of TBI, enhanced interactions between brain‐associated cells, especially astrocytes and oligodendrocyte precursor cells, were observed. Several signaling pathways, including EGF, CSF, MIF inflammatory factors as well as PSAP and PTN neurotrophic factor signaling were significantly enhanced after TBI. Lactate metabolism scores were elevated in the TBI group, especially in astrocytes. During the subacute phase, the frequency of intercellular communication increased but its intensity decreased. Astrocytes and oligodendrocyte precursor cells remained at high levels during both phases. PSAP signaling was closely associated with the subacute phase of TBI. Subsequently, NADH:ubiquinone oxidoreductase subunit B9 (*Ndufb9*) and cytochrome *c* oxidase subunit 8A (*Cox8a*) were identified as key players in lactate metabolism associated with TBI. *Ndufb9* and *Cox8a* showed a consistent upward trend in brain tissue following TBI with transcriptomic data.

**Conclusion:**

Lactate metabolism genes play an important role in TBI. These findings provide new insights into the cellular and molecular mechanisms following TBI.

## Graphical abstract

To identify TBI‐related cell types by analyzing single‐cell and bulk RNA‐seq data, and to further elucidate the role of lactate metabolism in TBI by functional analysis and molecular mechanism analysis, which will provide a theoretical basis for clinical diagnosis and treatment of the disease.

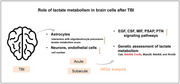

**Abbreviations**
aMG: activated microglia
AQP4: aquaporin 4
ASCs: astrocytes
BSCB: blood–spinal cord barrier
Calr: calreticulin
Cox8a: cytochrome *c* oxidase subunit 8A
DEGs: differentially expressed genes
END: endothelial
MAC: macrophages
MG: microglia
Mrps28: mitochondrial ribosomal protein S28
Ndufb8: NADH:ubiquinone oxidoreductase subunit B8
Ndufb9: NADH:ubiquinone oxidoreductase subunit B9
NEU: neurons
nODCs: new oligodendrocyte
ODCs: oligodendrocyte
OPCs: oligodendrocyte progenitor cells
PC: principal components
PCA: principal component analysis
PER: pericytes
Rrm2b: ribonucleotide reductase regulatory subunit M2B
scRNA‐seq: single‐cell RNA sequencing
SMCs: smooth muscle cells
TBI: traumatic brain injury

## 1 Introduction

Traumatic brain injury (TBI) is characterized by neurological impairment resulting from external mechanical forces. This condition often leads to persistent neurological and cognitive disabilities, and in severe cases, it can be fatal (Maas et al. 2022). Brain damage resulting from TBI can be classified into mild, moderate, or severe categories. It occurs instantaneously at the moment of impact as a primary injury and may further develop postimpact as a secondary injury (Maas et al. 2022; Tenovuo et al. 2021; Zibara et al. 2019). Mild to moderate TBIs account for about 90% of all TBI cases. TBI can result in neurological signs and cognitive dysfunction, substantially affecting life quality and necessitating substantial health service requirements (Cassidy et al. 2014; McAllister et al. 2006). Hence, the discovery of novel biomarkers for TBI can facilitate a deeper understanding of the pathological processes, improve diagnostic accuracy, enhance prognostic predictions, and foster the development of innovative treatments for TBI (Ghaith et al. 2022).

In the initial phase of TBI, energy demands surge due to impaired cerebral autoregulation and insufficient cerebral blood flow, leading to metabolic uncoupling and significant energy dysfunction (Carpenter et al. 2015). Cytotoxic edema is also rapidly triggered (Bordone et al. 2019). Cytotoxic edema as a premorbid process of pathologic cell swelling in TBI (Stokum et al. 2015). Water enters perivascular astrocytes (ASCs) along with an osmotic gradient (Tang and Yang 2016), causing them to swell (Simard et al. 2007) and disrupting the local osmotic environment. Lactate plays a crucial role in brain metabolism (Cai et al. 2022; Horvat et al. 2021). The activation of glycolysis by glutamate prompts the conversion of glucose to lactate in ASCs, which is then transported to NEU (Pellerin and Magistretti 1994). However, this view is somewhat controversial (Bak and Walls 2018). Studies have demonstrated that lactate production, which occurs not only through the glycolytic pathway but also, in certain instances, via the pentose phosphate pathway by transferring glucose 6‐phosphate, is elevated following an injury compared to the normal brain metabolism (Glenn et al. 2015). Should NEU be so impaired that they cannot effectively utilize the lactate supplied by ASCs, this scenario may lead to elevated levels of extracellular lactate (Carpenter et al. 2014). The cerebral tissue assimilates lactic acid that is generated in the periphery, designating lactic acid as an alternative fuel following TBI (Glenn et al. 2015). Nevertheless, the metabolic pathway of lactate following TBI warrants additional investigation.

Ability to probe cellular diversity and uncover the regulatory interactions between genes and cells that contribute to the pathophysiology of TBI has been enhanced by the evolution of single‐cell RNA sequencing (scRNA‐seq) techniques (Bolte et al. 2023; Jha et al. 2024). Arneson et al. (2022) reported comprehensive and novel molecular insights into the spatiotemporal gene regulation of TBI response and their connections to pathophysiological consequences. Despite these advancements, a delineation of the molecular intricacies and pathways associated with lactate metabolism in post‐traumatic conditions remains uncharted territory. In this study, scRNA‐seq and bulk RNA‐seq datasets were analyzed to identify cell types and interactions relevant to TBI. In addition, we shed light on the significance of lactate metabolism within TBI by conducting functional assessments coupled with analyses of molecular mechanisms, establishing a foundation for the clinical evaluation and therapeutic management of the disorder.

## 2 Materials and Methods

### 2.1 Data Sources

Samples of single‐cell data and transcriptome microarray data (GSE180862 and GSE128543) from TBI mouse downloaded from the GEO database (https://www.ncbi.nlm.nih.gov/geo/). In the GSE180862 (Arneson et al. 2022) scRNA‐seq dataset, 10‐week‐old male C57BL/6 J (B6) mice were randomly assigned to undergo either mild fluid percussion injury (TBI) or sham surgery, and researchers were not blinded. They performed scRNA‐seq analysis of the central nervous system (hippocampus and frontal cortex) and blood circulation (peripheral blood leukocytes) of mice treated or not treated with TBI during the acute (24 h) and subacute (7 days) periods. In this study, we used data from the frontal cortex tissue of mice. The GSE128543 dataset contains seven TBI samples and seven non‐TBI samples in cortical brain tissue, TBI was induced in 12–15 week old mice by controlled cortical impact injury, analyzed 48 h postinjury in C57BL/6 mice. We used the GSE128543 (Jackson et al. 2020) dataset to validate the results of the analysis of the GSE180862 data. This study incorporated 245 genes linked to lactate metabolism, which are derived from seven distinct lactate‐associated metabolic pathways (HP_INCREASED_SERUM_LACTATE, GOMF_LACTATE_TRANSMEMBRANE_TRANSPORTER_ACTIVITY, HP_ABNORMAL_LACTATE_DEHYDROGENASE_LEVEL, HP_INCREASED_LACTATE_DEHYDROGENASE_LEVEL, GOBP_LACTATE_TRANSMEMBRANE_TRANSPORT, and GOMF_L_LACTATE_DEHYDROGENASE_ACTIVITY) as outlined in the MSigDB database (Wang et al. 2022).

### 2.2 Single‐Cell Analysis and Cell‐Type Annotation

Normalization and dimensionality reduction of the original data of GSE180862 by the R package “Seurat” (Seth et al. 2022). Ineligible cells include genes that can only be detected in three or fewer cells and low‐quality cells with less than 200 genes detected will be excluded from subsequent analysis. The CellCycleScoring function is used to compute cell‐cycle scores, storing the S and G2/M scores and the predicted classification of each cell in G2/M, S, or G1 phase in the object metadata. In addition, the identity of the Seurat object is set to cell‐cycle staging via set.identity = TRUE. The FindVariableFeatures function was used to identify highly variable genes. Multiple samples were integrated using the R package “harmony” principal component analysis (PCA) was performed on single‐cell samples, and the top 20 principal components (PC) were selected for subsequent analysis. Cell‐type annotation was performed using R package “singleR” and marker in literature (Arneson et al. 2022). Finally, DimPlot and FeaturePlot functions in R package “Seurat” were used to plot marker gene bubbles, different cell types, and sample percentage.

### 2.3 Analysis of Cell Populations Related to Lactate Metabolism

The cellular activity scores of the lactate metabolism‐related gene set in the single‐cell transcriptome were determined using the R package “AUCell,” which also identified the specific cell types in the brain affected by lactate metabolism (Lu et al. 2021).

### 2.4 Analysis of Differential Enrichment

The differences between the whole cell and subpopulation of cells were analyzed using the FindMarkers function (min.pct = 0.25, logfc.threshold = 0.25) in the R package “Seurat,” and the heatmap of differential genes was plotted in the R package “pheatmap.” Heatmaps of differential genes were drawn using the R package “heatmap.” The functional enrichment of these differential genes was analyzed with the R package “clusterProfiler” and histograms as well as bubble plots of the functional enrichment results were created using the R package “ggplot2.”

### 2.5 Analysis of Transcription Factor

R package “SCENIC” performs transcription factor prediction on key cell populations (Van de Sande et al. 2020), highlights key transcription factors between different subgroups, and finds differential transcription factors between TBI and sham groups.

### 2.6 Analysis of Cellular Interactions

Analyze cellular communication between TBI and non‐TBI cells using the R package “CellChat” to find key receptors, ligands, and pathways, and to demonstrate the relationship between key cell populations (Jin et al. 2021). Ligand‐receptor pairs with a *p* value of less than 0.05 determined by CellChat were considered to be significant interacting molecules between different cell subpopulations. In addition, by constructing CellChat objects that recognize overexpressed genes and their ligand‐receptor interactions, CellChat is able to calculate the probability of cell‐to‐cell communication. Biologically meaningful cell–cell communication is inferred by assigning a probability value to each interaction and performing a permutation test.

### 2.7 Screening of TBI‐Related Lactate Metabolism Genes

Overlapping genes were taken for TBI acute injury (24 h) and TBI subacute injury (7 days) mechanism significant difference genes and lactate metabolism related genes to obtain TBI related lactate metabolism related genes in ASCs. R package “VennDiagram” draws Venn diagrams. Subsequently, it was verified that the expression levels of the key genes were present in the GSE128543 transcriptome data. Significant differences indicated using a *p* value of < 0.05. Next, the Bonferroni correction was applied to adjust the raw *p* value for the false discovery rate (Glickman et al. 2014). R package “ggplot2” plots gene expression box plots.

### 2.8 Statistics

All data processing and analytical procedures were conducted using the R (v4.3.3). Comparative analysis between the two sample groups was executed utilizing either the Wilcoxon rank‐sum test or the *t*‐test. *p* < 0.05 was considered to be statistically significantly different.

## 3 Results

### 3.1 scRNA‐Seq Data Analysis Identifies TBI Cell Subtypes

**FIGURE 1:**
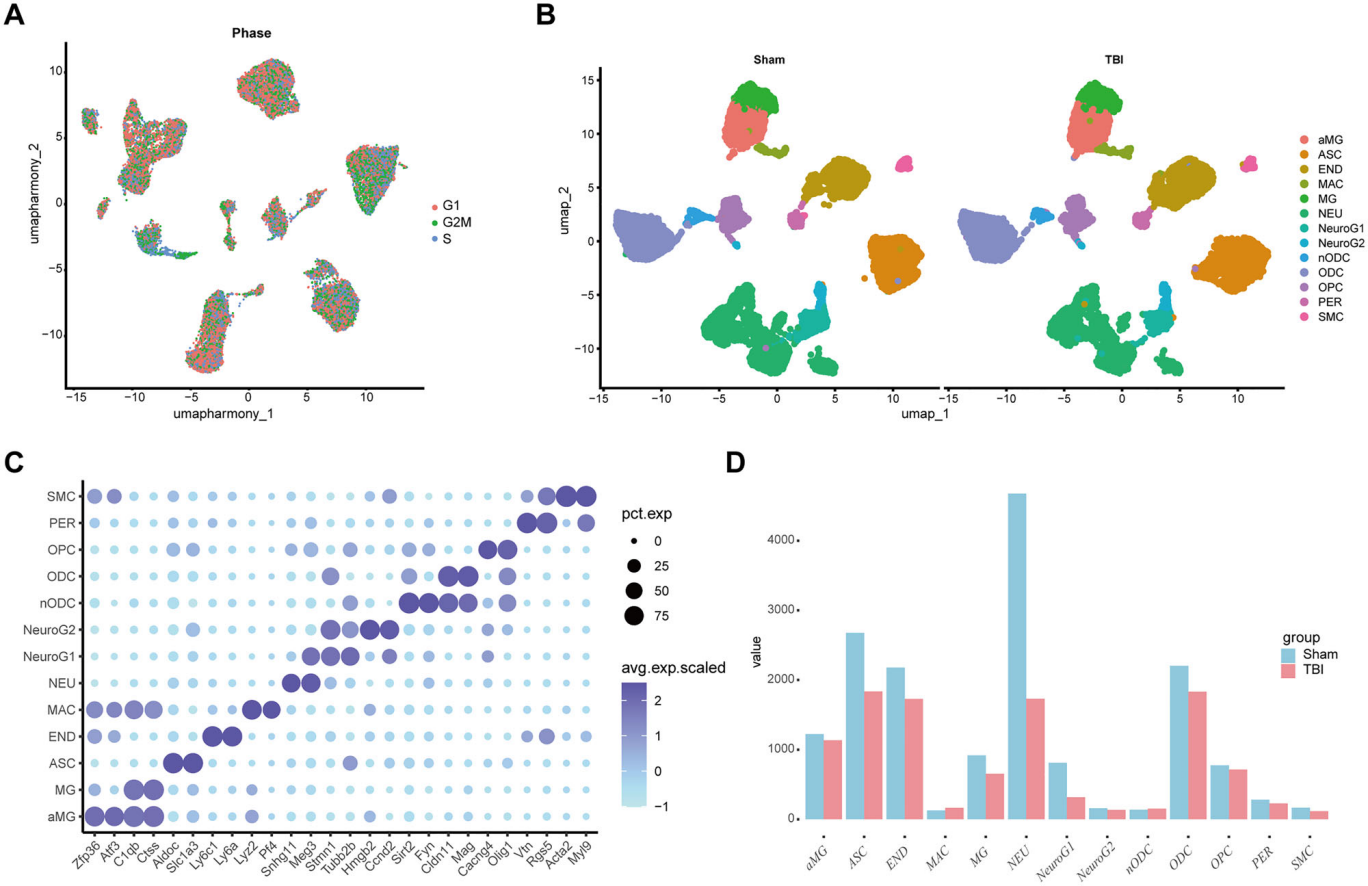
ScRNA‐seq data identifying the major cell types in acute and subacute mouse frontal cortex tissues after TBI. (A) UMAP plot of cell cycle‐related gene expression. (B) UMAP plots of the distribution of all cell types in the TBI and sham groups. (C) Bubble plots of the expression of marker genes per cell type. (D) Histogram of the number of cells in different groups. aMG, activated microglia; ASC, astrocytes; END, endothelial; MAC, macrophages; MG, microglia; NEU, neurons; nODCs, new oligodendrocyte; ODCs, oligodendrocyte; OPCs, oligodendrocyte progenitor cells; PER, pericytes; SMCs, smooth muscle cells; TBI, traumatic brain injury.

To pinpoint transcriptional alterations in cell types related to TBI, a total of 16,336 cells from the sham group and 10,745 cells from the TBI group were examined, sourced from mouse frontal cortex tissues within the publicly available scRNA‐seq dataset GSE180862 (Arneson et al. 2022). The cell cycle phase (G1, S, or G2/M phase) that each single cell is in was first assessed. This is essential for understanding the functional state and physiological properties of the cells. The PCA of cycle genes was downscaled and the effect of cell cycle genes on cell clustering was viewed. The cell cycle had essentially no effect on cell clustering in this dataset (Figure 1A). Identification and visualization of distinct cell populations in acute and subacute samples by UMAP downscaling technique (Figure 1B). Altogether, 13 cell populations were distinguished, including neurons (NEU), NeuroG1, macrophages (MAC), pericytes (PER), ASCs, new oligodendrocyte (nODCs), oligodendrocyte (ODCs), activated microglia (aMG), endothelial (END), NeuroG2, microglia (MG), oligodendrocyte progenitor cells (OPCs), and smooth muscle cells (SMCs). The expression levels of cell‐type markers were plotted to verify the precision of cell‐type classifications (Figure 1C). In addition, the variations in cell counts between the TBI group and the sham group were evaluated. A reduction in the number of cells such as NEU, ASC, and END cells was exhibited by the TBI group when compared to the sham group (Figure 1D).

### 3.2 Analysis of Acute Related Processes in TBI

**FIGURE 2:**
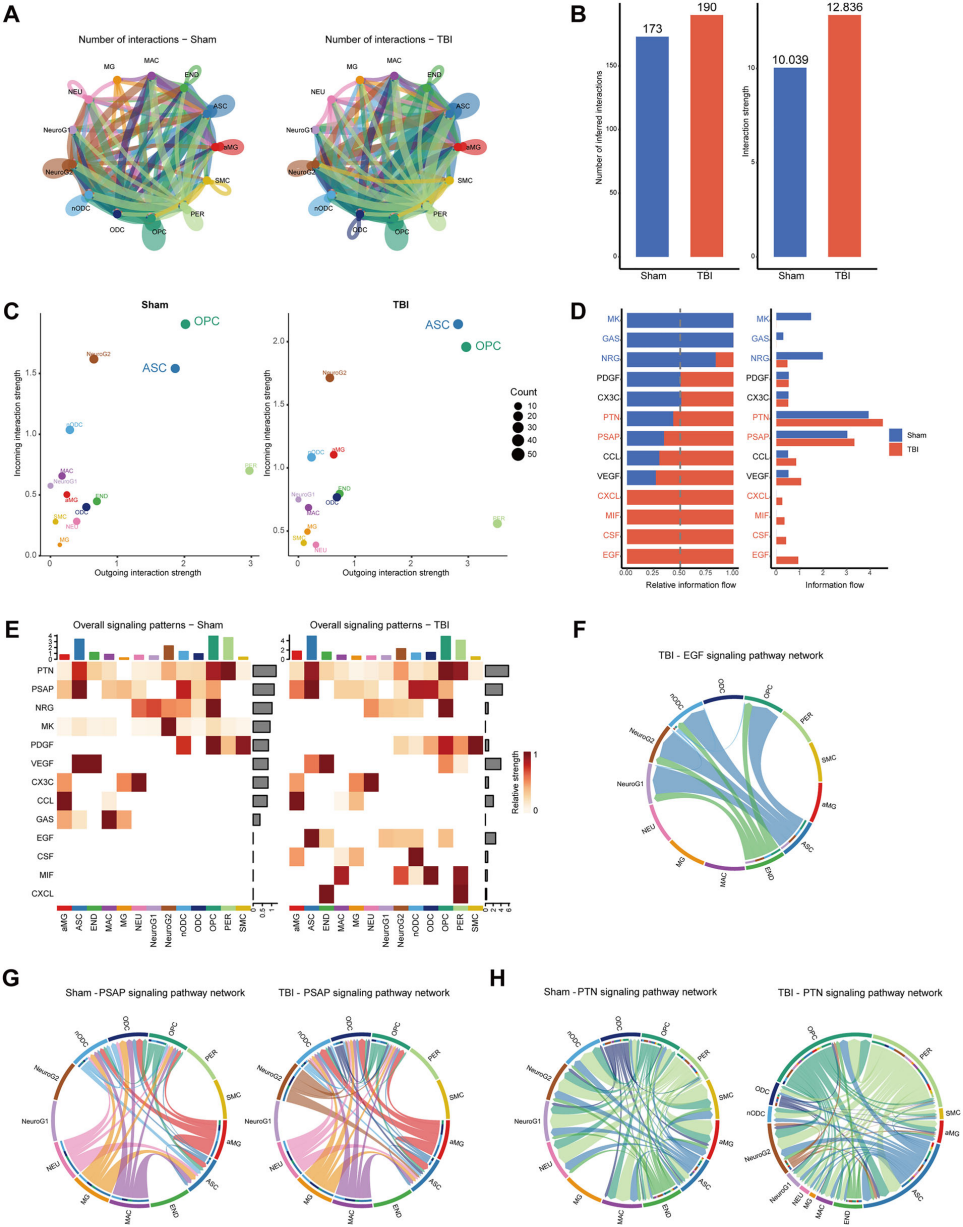
Intercellular communication networks after acute effects of TBI. (A) Number and strength of cellular interactions in the TBI and sham groups. (B) Histogram of differences in the number and strength of cellular interactions between the TBI and sham groups. (C) Bubble plots of changes in the strength of afferent and efferent interactions in different cell types after TBI. (D) Changes in relative signaling after TBI. (E) Cell‐signaling pathway correlations in TBI and sham groups. (F) EGF‐related signaling pathway network in acute TBI. (G) Acute TBI and sham group PSAP‐related signaling pathway network. (H) Acute TBI and sham group PTN‐related signaling pathway network. aMG, activated microglia; ASC, astrocytes; END, endothelial; MAC, macrophages; MG, microglia; NEU, neurons; nODCs, new oligodendrocyte; ODCs, oligodendrocyte; OPCs, oligodendrocyte progenitor cells; PER, pericytes; SMCs, smooth muscle cells; TBI, traumatic brain injury.

Brain injury can lead to shock coma, among other things (Lin et al. 2022). A comparative analysis of 24 h samples of brain injury was performed to determine the effects of acute injury. The brain cells are closely interconnected, and comparatively speaking, brain injury leads to a higher total number of cell–cell interactions and a greater intensity of interactions (Figure 2A,B). Furthermore, after TBI, ASCs were the cell population that had significant changes in sending or receiving signals (Figure 2C). Several signaling pathways were significantly enhanced after TBI, including EGF, CSF, inflammatory MIF, and atypical neurotrophic factor (PSAP and PTN) signaling (Figure 2D,E). ASCs and END cells express EGF, a signaling pathway that protects NEU (Figure 2F). Almost all cells secrete PSAP and PTN (Figure 2G,H).

**FIGURE 3:**
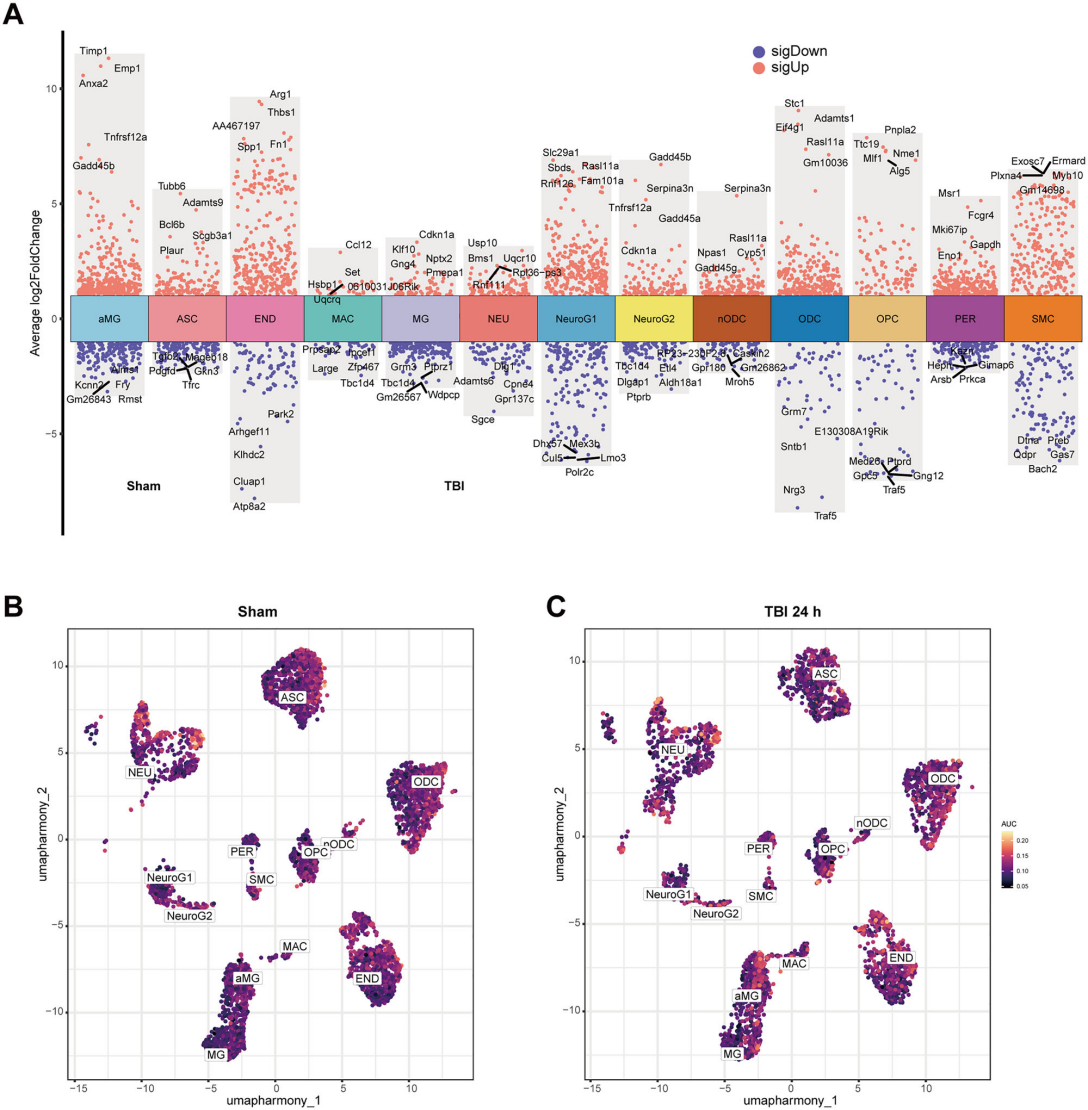
Analysis of differences and AUCell analysis related to lactate metabolism in acute TBI. (A) Top five DEGs with log2FC values for each single‐cell subpopulation after analysis of differences between sham and TBI groups. (B, C) AUCell scores of sham and TBI groups of cells in the set of genes related to lactate metabolism. aMG, activated microglia; ASC, astrocytes; DEGs, differentially expressed genes; END, endothelial; MAC, macrophages; MG, microglia; NEU, neurons; nODCs, new oligodendrocyte; ODCs, oligodendrocyte; OPCs, oligodendrocyte progenitor cells; PER, pericytes; SMCs, smooth muscle cells; TBI, traumatic brain injury.

The “FindAllMarkers” algorithm was employed to analyze differentially expressed genes (DEGs) within each cell population, and a total of 10,363 DEGs were screened (Figure 3A). In addition, we compared the AUCell scores of sham and TBI 24 h groups cells in genomes related to lactate metabolism (Figure 3B,C).

### 3.3 Analysis of Subacute Related Processes in TBI

**FIGURE 4:**
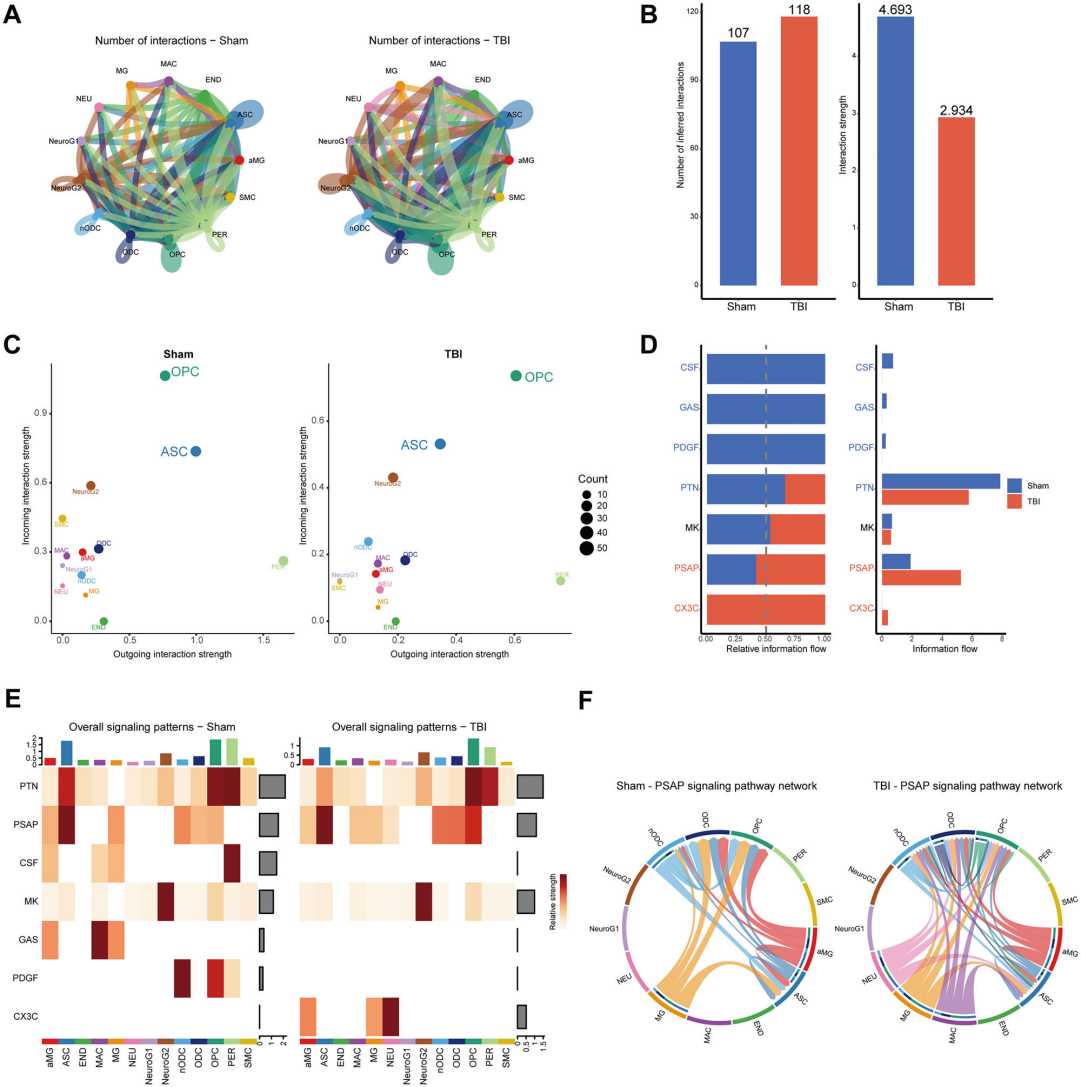
Intercellular communication networks after subacute effects of TBI. (A) Number and strength of cellular interactions in the subacute TBI and sham groups. (B) Histogram of differences in the number and strength of cellular interactions between the subacute TBI and sham groups. (C) Bubble plots of changes in the strength of afferent and efferent interactions in different cell types after subacute TBI. (D) Changes in relative signaling after subacute TBI. (E) Cell‐signaling pathway correlations in subacute TBI and sham groups. (F) Subacute TBI and sham group PSAP‐related signaling pathway network. aMG, activated microglia; ASC, astrocytes; END, endothelial; MAC, macrophages; MG, microglia; NEU, neurons; nODCs, new oligodendrocyte; ODCs, oligodendrocyte; OPCs, oligodendrocyte progenitor cells; PER, pericytes; SMCs, smooth muscle cells; TBI, traumatic brain injury.

We similarly investigated the cell types whose intercellular communication was affected during the subacute (7 days) phase of TBI (Ji et al. 2023). During the subacute phase of TBI, an increase in the frequency of intercellular communications was observed, but the strength of these communications was diminished (Figure 4A,B). Notably, incoming interaction strength of ASCs and OPCs remains high in both the subacute phases of TBI (Figure 4C). A comparison of the reciprocal signaling pathways between the TBI and the sham groups indicated that the PSAP signaling pathway was intimately associated with the subacute phase of TBI (Figure 4D,E). PSAP signaling was enhanced after TBI (Figure 4F).

**FIGURE 5:**
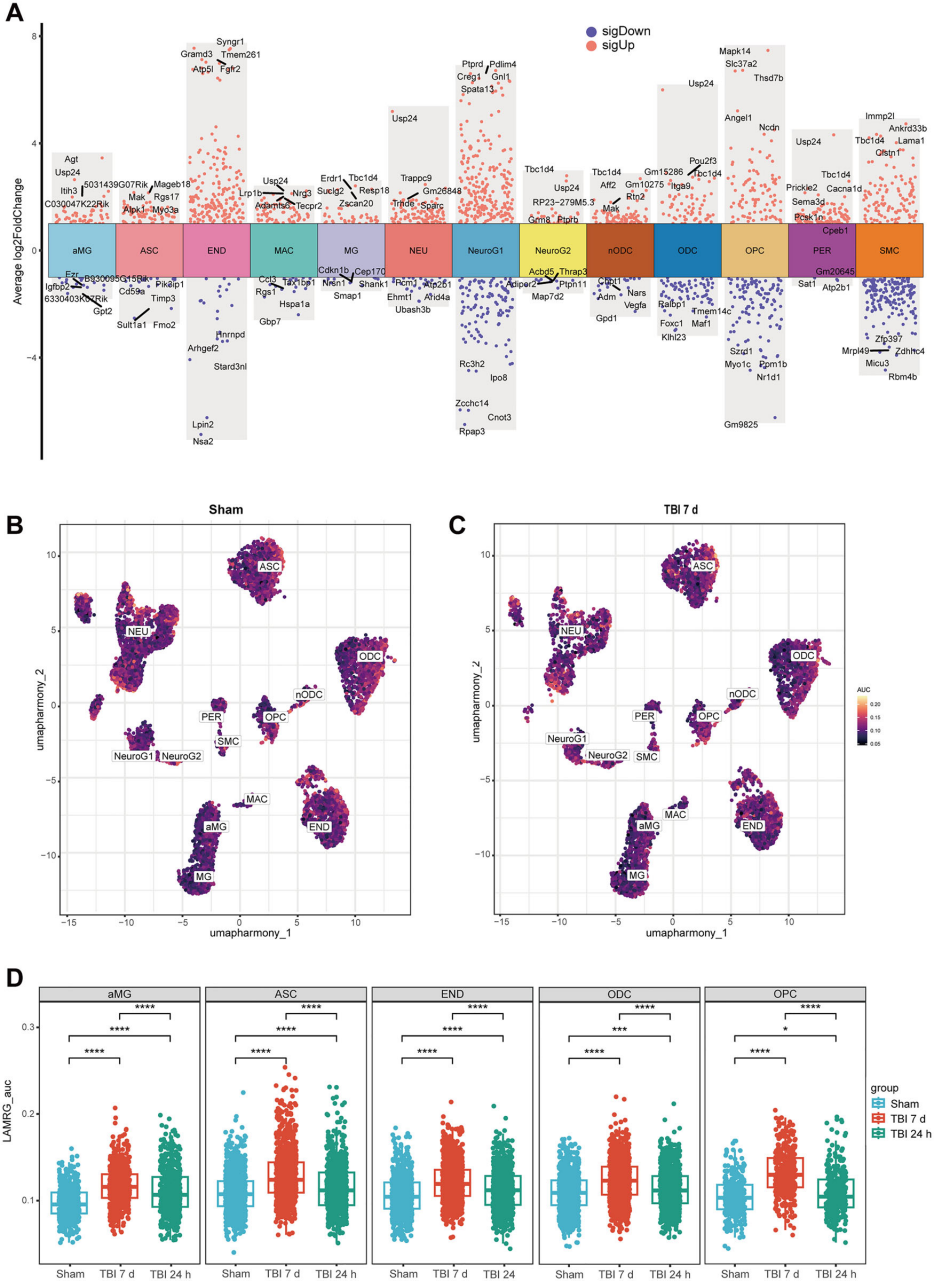
Analysis of differences and AUCell analysis related to lactate metabolism in subacute TBI. (A) Top five DEGs with log2FC values for each single‐cell subpopulation after analysis of differences between sham and subacute TBI groups. (B, C) Distribution of AUCell scores for the sham (B) and subacute TBI (C) groups. aMG, activated microglia; ASC, astrocytes; DEGs, differentially expressed genes; END, endothelial; MAC, macrophages; MG, microglia; NEU, neurons; nODCs, new oligodendrocyte; ODCs, oligodendrocyte; OPCs, oligodendrocyte progenitor cells; PER, pericytes; SMCs, smooth muscle cells; TBI, traumatic brain injury.

During the subacute phase of TBI, numerous cellular transcriptomes were impacted. We screened 5985 DEGs in both groups and also identified the top five genes with log2FC values (Figure 5A). The AUCell overall score profile for the gene set related to lactate metabolism, which was similarly distributed in both the TBI 7 days and sham groups (Figure 5B,C). However, in aMGs, ASCs, ENDs, ODCs, and OPCs, we observed that the lactate metabolism AUCell scores were significantly higher than those of the sham group at 24 h and 7 days after TBI (Figure 5D). In addition, lactate scores were significantly higher in these cells at 7 days of TBI compared to 24 h of TBI (Figure 5D). Expression of lactate‐related genes was consistently increased during the acute and subacute phases of TBI in most brain cells.

### 3.4 ASCs‐Lactate Metabolism Interaction and Transcriptional Regulation Following TBI

ASCs demonstrate robust cellular interactions post‐TBI, affecting principal signaling pathways associated with major neurological diseases (Alhadidi et al. 2024). Furthermore, the AUCell scores for the lactate metabolism‐related gene set varied between the sham and TBI groups. Consequently, the subsequent study focused on the relationship between ASCs and lactate metabolism.

**FIGURE 6:**
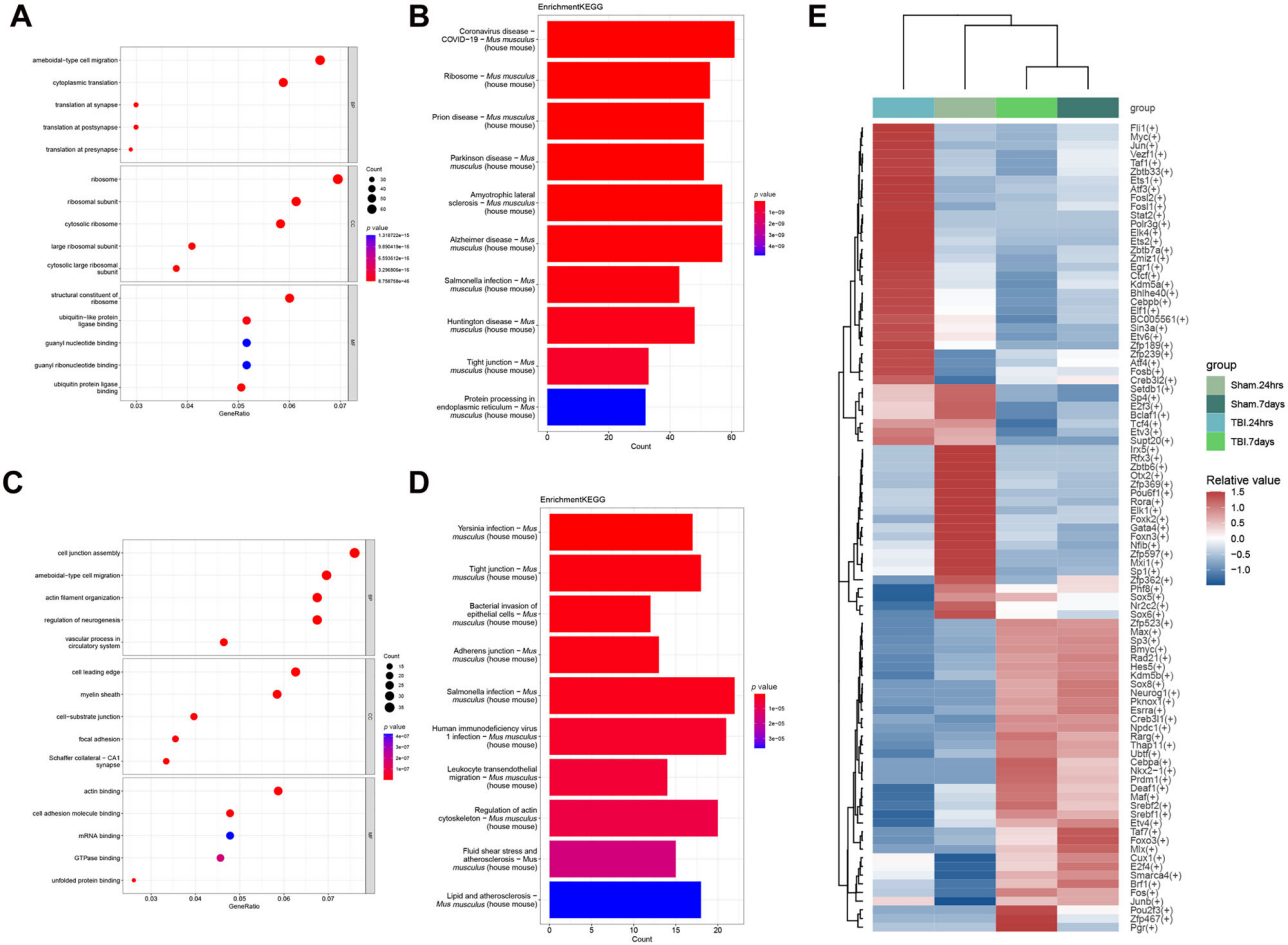
Functional enrichment and transcription factor analysis of DEGs. (A, B) Acute brain injury astrocyte differential gene GO enrichment map and KEGG pathway histogram. (C, D) Differential gene GO enrichment and KEGG pathway histogram of astrocytes from subacute brain injury. (E) Heatmap of key transcription factors in different groups of ASCs. aMG, activated microglia; ASC, astrocytes; END, endothelial; GO, gene ontology; KEGG, Kyoto Encyclopedia of Genes and Genomes; MAC, macrophages; MG, microglia; NEU, neurons; nODCs, new oligodendrocyte; ODCs, oligodendrocyte; OPCs, oligodendrocyte progenitor cells; PER, pericytes; SMCs, smooth muscle cells.

During the acute phase of TBI, GO and KEGG enrichment analyses were performed on DEGs in ASCs. Go analyses showed enrichment of ameboidal‐type cell migration and ribosome signaling pathways (Figure 6A), and KEGG results showed enrichment of pathways related to protein processing in endoplasmic reticulum and pathways related to brain diseases, particularly the tight junction proteins‐related pathway, since they are part of the blood–brain barrier (Lochhead et al. 2020), which is affected in TBI and its disruption can lead to long‐term consequences in the brain (Figure 6B). In the subacute phase, GO enrichment analysis of DEGs in ASCs showed enrichment of GTPase‐binding and actin cytoskeleton‐related pathways (Figure 6C), and KEGG enrichment analysis showed enrichment of the Leukocyte transendothelial migration pathway (Figure 6D). On the other hand, we demonstrated that TBI affects gene expression levels by transcription factor analysis, which revealed that different groups were regulated by different transcription factors (Figure 6E).

### 3.5 Lactate Metabolism Gene Signature in ASCs Post‐TBI

**FIGURE 7:**
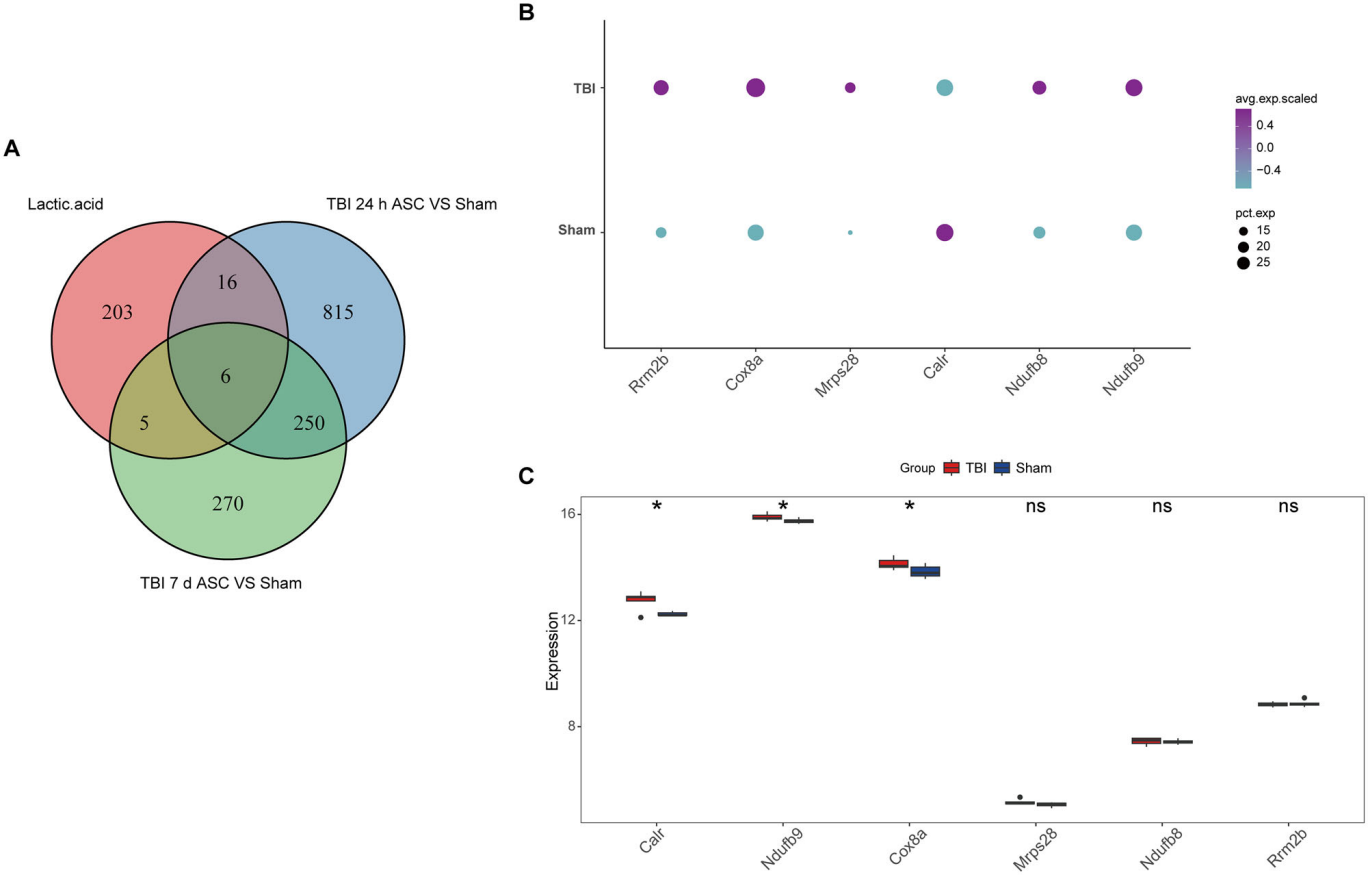
Identification of genes related to lactate metabolism. (A) Venn diagram of astrocyte DEGs and genes related to lactate metabolism in two brain injury mechanisms. (B) Expression bubble plot of six key genes between the sham and TBI groups. (C) Boxplots of the expression of six key genes in brain tissue from TBI and controls in the GSE128543 dataset. aMG, activated microglia; ASC, astrocytes; END, endothelial; MAC, macrophages; MG, microglia; NEU, neurons; nODCs, new oligodendrocyte; ODCs, oligodendrocyte; OPCs, oligodendrocyte progenitor cells; PER, pericytes; SMCs, smooth muscle cells; TBI, traumatic brain injury. *p < 0.05, **p < 0.01, ***p < 0.001, and “ns” represents non‐significant.

Six lactate metabolism genes (calreticulin [*Calr*], NADH:ubiquinone oxidoreductase subunit B9 [*Ndufb9*], cytochrome *c* oxidase subunit 8A [*Cox8a*], mitochondrial ribosomal protein S28 [*Mrps28*], NADH:ubiquinone oxidoreductase subunit B8 [*Ndufb8*], and ribonucleotide reductase regulatory subunit M2B [*Rrm2b*]) were identified by the intersection of lactate metabolism‐related genes with the two injury mechanisms of TBI (Figure 7A). Meanwhile, the acute injury mechanism was more related to lactate metabolism with more intersecting genes. *Ndufb9*, *Cox8a*, *Mrps28*, *Ndufb8*, and *Rrm2b* were highly expressed in the TBI group, while *Calr* was highly expressed in the sham group (Figure 7B). Out of these, the difference between these six genes (TBI vs. sham) remained significant after Bonferroni correction in the acute and subacute phases of TBI in ASC (Tables S1 and S2). In addition, we validated these genes using the transcriptomic dataset GSE128543 and found that *Ndufb9* and *Cox8a* were expressed at significantly higher levels in brain tissue after TBI (Figure 7C). However, this finding was not considered significant after Bonferroni correction of *p* value (Table S3). This reinforces the importance of these genes in the pathologic process of TBI and suggests that they may be potential therapeutic targets or disease markers.

## 4 Discussion

ScRNA‐seq allows us to discover new cell types, cell–cell interactions, and signaling pathways under pathological conditions, offering fresh insights for the development of therapeutic strategies (Cardona‐Alberich et al. 2021; Su et al. 2024; Tsai et al. 2023). In this research, we examined scRNA‐seq data from the frontal cortex and identified 13 distinct cell types, including NEU, ASCs, and MG. We also uncovered alterations in intercellular communication linked to the acute and subacute stages of TBI. Analysis of cellular interactions with lactate metabolism scores revealed that acute TBI is more highly correlated with lactate metabolism and induces inflammation‐related functions. We focused on the role of ASCs in TBI and found a strong association between lactate metabolism and ASCs, screening for two lactate metabolism genes, *Ndufb9* and *Cox8a*, in ASCs that would be affected by two mechanisms of TBI injury.

The decline in the number of END, ASCs, and NEU cells in the TBI group indicates that these cell types might be more vulnerable to injury or death during TBI. The change in cell number may be closely related to the decline in neurologic function after TBI (Akamatsu and Hanafy 2020). It is known that ASCs play a crucial role in both acute and chronic responses to mTBI, with the resultant changes in gene expression, morphology, proliferation, and function being referred to as ASCs proliferation (Burda et al. 2016; Shandra et al. 2019; Shinozaki et al. 2017). ASCs regulate brain homeostasis and the extracellular environment (Ransom and Ransom 2012). Analysis of intercellular communication showed that MG and ASCs were more affected in both the acute and subacute phases of brain injury. In particular, ASCs exhibit a high degree of connectivity with cell types such as NEU. ASCs interact with all cellular elements of the CNS, and a single ASC can connect with up to 2 million neuronal synapses in the human brain (Halassa et al. 2007; B. Zhou et al. 2019). These neuronal populations could be notably vulnerable to metabolic inhibition, and ASCs might play a role in stabilizing or inhibiting neuronal circuit function following TBI (Arneson et al. 2022). Furthermore, the PSAP signaling pathway has a strong association with TBI, evident in both the acute and subacute phases.

Within the frontal cortex, during both the acute and subacute stages, we noted significant shifts in the comprehensive transcriptomic profile of ASCs, characterized by close gene‐to‐gene transcriptional synchronization in the aftermath of trauma. We identified six genes (*Calr*, *Ndufb9*, *Cox8a*, *Mrps28*, *Ndufb8*, and *Rrm2b*) associated with lactate metabolism in ASCs. Notably, the expression levels of these genes were generally elevated in the TBI group compared to the sham group, with *Ndufb9* and *Cox8a* showing particularly pronounced increases. *Ndufb9* is associated with mitochondrial respiratory chain complex I assembly (Vera‐Montecinos et al. 2021). The dysfunction of complex I, which is a key component of the electron transport chain in cellular respiration, plays a significant role in oxidative phosphorylation disorders. *Ndufb9* has been reported to be a prognostic biomarker for uveal melanoma (Choi et al. 2020), breast cancer (Li et al. 2015), and osteosarcoma (P. Zhou et al. 2024). *Cox8a* enable cytochrome c oxidase activity, located in mitochondrion (Rotko et al. 2021). Deletion of *Cox8a* leads to a decrease in the cytochrome c oxidase protein complex and its enzymatic activity, with implications for cytochrome c oxidase enzyme stability (Rotko et al. 2021). Within the middle cerebral artery occlusion mouse model, a distinct cluster of cells was identified, characterized by an active respiratory electron transport chain and oxidative phosphorylation, notably expressing *Cox8a* (Zheng et al. 2022). This result laterally confirms a possible association between *Cox8a* and TBI. In addition, *Mrps28*, encoding mitochondrial ribosomal protein S28, has been shown to be affected in its expression after mild TBI (Zhang et al. 2024). The mechanism of action of *Calr*, *Ndufb8*, and *Rrm2b* in the post‐TBI brain is unclear. This implies that lactate metabolism could play a significant role in ASCs following TBI, possibly involving reprogramming of energy metabolism or the function of lactate as a signaling molecule.

Regulation of ASCs volume has been shown to be a key factor in the devastating consequences of CNS edema after TBI (Vella et al. 2015). In cytotoxic edema, ASCs swelling is caused by increased intracellular osmotic pressure due to ATP depletion and influx of extracellular ions (e.g., Na^+^ and Cl^−^) along with their electrochemical gradients, which drives water flux into ASCs without immediately affecting brain tissue volume. Aquaporin 4 (AQP4) plays an important role in the development of cytotoxic edema and is abundantly expressed in ASCs (Oklinski et al. 2014). That study shows that CNS edema is associated with increases both in total AQP4 expression and AQP4 subcellular translocation to the blood–spinal cord barrier (BSCB). Pharmacological inhibition of AQP4 translocation to the BSCB prevents the development of CNS edema and promotes functional recovery in injured rats (Kitchen et al. 2020). It has been shown that AQP4 continuously cycles between the cell surface, Rab5‐positive early and Rab11‐positive recirculating endosomes in mammalian cells. Internalization of AQP4 is kinesin‐dependent and AQP4 transport mechanisms are impaired upon inhibition of Rab5 and Rab11 function and cytoskeletal dynamics, which reveals a potential therapeutic target for edema therapy (Markou et al. 2024). This role has recently been confirmed by the work of Sylvain et al. (2021) which has demonstrated that targeting AQP4 effectively reduces cerebral edema during the early acute phase in stroke using photothrombotic stroke model. They have also shown a link to brain energy metabolism as indicated by the increase of glycogen levels. Furthermore, ASCs in the progression of brain injury and neurodegenerative diseases exhibit complex and diverse roles for AQP4. Studies using mice with specific AQP4 deletion in ASCs have also revealed a protective effect in the acute phase of stroke or TBI, reducing cytotoxic edema (Haj‐Yasein et al. 2011; Markou et al. 2022; Yao et al. 2015). However, into the chronic phase or recovery period, changes in AQP4 expression are more often associated with edema regression and neurological recovery. For example, in the rat model, AQP4 expression was negatively correlated with the degree of edema, and its upregulation was consistent with the regression of edema as measured by MRI in vivo (Fukuda et al. 2012). Thus, AQP4 ASCs play multiple roles at different stages of the disease, with both potentially deleterious and significant protective effects, which provide important clues for understanding their pathophysiological mechanisms and developing targeted therapeutic strategies. Given that lactate metabolism and AQP4‐mediated water transport are intricately linked to astrocyte function and CNS edema formation, it is plausible that the metabolic reprogramming observed in ASCs post‐TBI could influence the efficacy of therapeutic strategies aimed at reducing edema.

While this study provides valuable insights into the cellular and molecular alterations in TBI, several limitations should be noted. First, the analysis is based on mouse models, which may not fully recapitulate the complexity and heterogeneity of human TBI. Second, despite the potential promise of glial cells as therapeutic targets, the issue of drug specificity must be carefully considered in practical applications. Finally, while the lactate metabolism gene signature identified in ASCs is intriguing, further validation in larger, more diverse cohorts and functional studies are needed to fully understand its role in TBI and its potential as a therapeutic target.

In summary, we analyzed the network of cell‐to‐cell interactions between 13 cell types in acute and subacute phase TBI, and involved analyzing transcriptional disparities and gene sets pertaining to lactate metabolism across various cell types. The genes *Ndufb9* and *Cox8a*, which are associated with lactate metabolism, exhibit upregulation in ASCs within the TBI cohort. These genes are influenced by acute and subacute mechanisms of injury inherent to TBI. Our findings provide potential therapeutic targets for TBI intervention.

## Author Contributions

**Zhang Bu**: conceptualization, methodology, software, writing – original draft, writing – review and editing. **Yuqian Zhou**: data curation, writing – review and editing. **Feng Xu**: validation, writing – review and editing. **Shan Xu**: supervision, investigation, writing – review and editing.

## Ethics Statement

The authors have nothing to report.

## Consent

The authors have nothing to report.

## Conflicts of Interest

The authors declare no conflicts of interest.

### Peer Review

The peer review history for this article is available at https://publons.com/publon/10.1002/brb3.70428.

## Supporting information



Supporting Information

## Data Availability

The datasets supporting the conclusions of this article are included within the article.
