# Supplementary material for: Single‐Cell RNA and Transcriptome Sequencing to Analyze the Role of Lactate Metabolism in Traumatic Brain Injury Astrocytes
Source: Brain Behav. 2025 May 21;15(5):e70428. doi: 10.1002/brb3.70428 (PMC12092974; doi:10.1002/brb3.70428)
Supplement: Supplementary file 1 — Supporting Information [file BRB3-15-e70428-s001.docx]

**Supplementary table 1 In ASC, the differential p-value of six genes (TBI 24 h vs. Sham)**

| Gene | Raw.p | Bonferroni |
| --- | --- | --- |
| Ndufb8 | 0.000752142619234738 | 0.00451285571540843 |
| Ndufb9 | 0.000911435816274099 | 0.00546861489764459 |
| Rrm2b | 1.07442078033414e-12 | 6.44652468200484e-12 |
| Mrps28 | 1.50832721413098e-06 | 9.04996328478588e-06 |
| Calr | 4.32126849323803e-05 | 0.000259276109594282 |
| Cox8a | 6.45532403523205e-08 | 3.87319442113923e-07 |

**Supplementary table 2 In ASC, the differential p-value of six genes (TBI 7 d vs. Sham)**

| Gene | Raw.p | Bonferroni |
| --- | --- | --- |
| Ndufb9 | 0.000715285002823807 | 0.00429171001694284 |
| Cox8a | 0.0258696476076388 | 0.155217885645833 |
| Ndufb8 | 0.0285824544534193 | 0.171494726720516 |
| Calr | 0.0485706785837773 | 0.291424071502664 |
| Rrm2b | 1.40415474599368e-44 | 8.42492847596208e-44 |
| Mrps28 | 6.88120115083279e-30 | 4.12872069049967e-29 |

**Supplementary table 3 In GSE128543, the differential p-value of six genes (TBI vs. Sham)**

| Gene | Raw.p | Bonferroni |
| --- | --- | --- |
| Calr | 0.000235929507678131 | 0.00141557704606879 |
| Cox8a | 0.0145550242812495 | 0.087330145687497 |
| Ndufb9 | 0.0270625164829814 | 0.162375098897888 |
| Mrps28 | 0.114202086907839 | 0.685212521447034 |
| Rrm2b | 0.595384122831976 | 1 |
| Ndufb8 | 0.656996710552981 | 1 |
